# Deciphering the Interplay between the Epithelial Barrier, Immune Cells, and Metabolic Mediators in Allergic Disease

**DOI:** 10.3390/ijms25136913

**Published:** 2024-06-24

**Authors:** Lea Ling-Yu Kan, Peiting Li, Sharon Sze-Man Hon, Andrea Yin-Tung Lai, Aixuan Li, Katie Ching-Yau Wong, Danqi Huang, Chun-Kwok Wong

**Affiliations:** 1 Institute of Chinese Medicine, State Key Laboratory of Research on Bioactivities and Clinical Applications of Medicinal Plants, The Chinese University of Hong Kong, Hong Kong, China; lea-kan@cuhk.edu.hk (L.L.-Y.K.); peiting@link.cuhk.edu.hk (P.L.); andrealai@link.cuhk.edu.hk (A.Y.-T.L.); 2 Department of Chemical Pathology, The Chinese University of Hong Kong, Prince of Wales Hospital, Hong Kong, China; sharonhon@link.cuhk.edu.hk (S.S.-M.H.); katiecywong@cuhk.edu.hk (K.C.-Y.W.); 1155171999@link.cuhk.edu.hk (D.H.); 3 School of Biomedical Sciences, The Chinese University of Hong Kong, Hong Kong, China; 1155181868@link.cuhk.edu.hk; 4 Li Dak Sum Yip Yio Chin R & D Centre for Chinese Medicine, The Chinese University of Hong Kong, Hong Kong, China

**Keywords:** allergy, eosinophils, ILC2, lipid mediators, allergic inflammation, metabolites

## Abstract

Chronic exposure to harmful pollutants, chemicals, and pathogens from the environment can lead to pathological changes in the epithelial barrier, which increase the risk of developing an allergy. During allergic inflammation, epithelial cells send proinflammatory signals to group 2 innate lymphoid cell (ILC2s) and eosinophils, which require energy and resources to mediate their activation, cytokine/chemokine secretion, and mobilization of other cells. This review aims to provide an overview of the metabolic regulation in allergic asthma, atopic dermatitis (AD), and allergic rhinitis (AR), highlighting its underlying mechanisms and phenotypes, and the potential metabolic regulatory roles of eosinophils and ILC2s. Eosinophils and ILC2s regulate allergic inflammation through lipid mediators, particularly cysteinyl leukotrienes (CysLTs) and prostaglandins (PGs). Arachidonic acid (AA)-derived metabolites and Sphinosine-1-phosphate (S1P) are significant metabolic markers that indicate immune dysfunction and epithelial barrier dysfunction in allergy. Notably, eosinophils are promoters of allergic symptoms and exhibit greater metabolic plasticity compared to ILC2s, directly involved in promoting allergic symptoms. Our findings suggest that metabolomic analysis provides insights into the complex interactions between immune cells, epithelial cells, and environmental factors. Potential therapeutic targets have been highlighted to further understand the metabolic regulation of eosinophils and ILC2s in allergy. Future research in metabolomics can facilitate the development of novel diagnostics and therapeutics for future application.

## 1. Introduction

Bioactive metabolites are part of a group of intermediary chemicals that play fundamental roles in cell signaling and processing, such as waste removal, energy production and expenditure, and nutrient transport. These molecular components are typically smaller than 1500 kDa and include carbohydrates, fatty acids, amino acids, and nucleic acids [1]. Recent technological advances and the availability of big data analytics have led to the discovery that metabolites participate in intermediatory signaling and energy metabolism and influence various physiological processes. These include the maintenance of homeostasis, defense against infectious diseases, and regulation of protein and cellular activities [2,3]. The biological system is sensitive to changes in the internal and external environment. Therefore, perturbations that occur at local and systemic levels result in tissue remodeling and metabolic abnormalities, ultimately leading to disease development [4,5,6]. While studies on the deregulation of cellular metabolism have been focused on obesity, diabetes, and cancer, there is growing interest in immunological diseases.

The maintenance of the immune system requires substantial energy and precise regulatory mechanisms to effectively strike a balance between tolerogenic and immunogenic responses in foreign or self-antigens. Allergic disease, commonly referred to as Type I hypersensitivity, arises from a dysfunctional immunological state in which foreign allergens such as house dust mites (HDMs) that are harmless to most individuals prompt a hyperreactive immune response [7]. The onset of allergy is likely caused by environmental factors, epigenetics, genetics, and microecology during early childhood [8]. Firstly, sensitization occurs when primary exposure to the antigen primes T cells and activates B cells to produce Immunoglobulin E (IgE) antibodies. Subsequent exposures to the same allergens trigger an inflammatory response, causing symptoms such as anaphylaxis, dermatitis, and asthma [9]. The T-helper type 2 (Th2) response is the primary inflammatory mechanism of IgE-mediated allergic reactions. It involves the secretion of cytokines IL-4, IL-5, IL-13, and IL-9, subsequently activating allergy-associated innate immune cells such as eosinophils, basophils, and mast cells [10]. In addition to changes in cellular morphology and the function of activated immune cells during inflammation, metabolic reprogramming alters the cellular metabolism and energy output [11,12].

Current technology enables the combined application of omics technologies, computational modeling, and modern experimental technology to carry out metabolic profiling and analyses. This provides new insights into linking cellular metabolic pathways and phenotypes between normal and disease states and facilitates drug discovery [13,14]. Immunometabolism is a study that combines metabolism and immunology, which is fundamental in discovering new crosstalk mechanisms between cells. Extensive research has been conducted on the metabolic characterization of basophils, macrophages, and T cells in allergic diseases. However, there is still a lack of understanding of the metabolic regulation of other common innate immune cells, their crosstalk, and their roles in chronic allergic inflammation. To elucidate the metabolic mechanisms in eosinophils and ILC2s, this review discusses how both cell types contribute to the pathogenesis of allergies and the ways in which metabolites shape interactions and functions in these cells, particularly through fatty acid metabolism. The potential role of metabolites in the therapeutic application of allergy will also be discussed.

## 2. Epithelial Barrier Theory and Immune Regulation

### 2.1. Epithelial Barrier Hypothesis

The exposome is defined as the total environmental exposures of an individual throughout their lifetime, in which certain environments can increase or decrease their susceptibility to immunological, metabolic, and autoimmune disorders [15,16]. According to the biodiversity hypothesis, increased environmental exposure may lead to a reduced risk of impaired immune response [17]. Population-based studies have shown that early exposure to agricultural environments is associated with lower incidence and prevalence of atopic sensitization in children [18,19]. This host-protective effect is likely related to a variety of microbial, fungal, and endotoxin exposures from farm milk consumption and encounters with livestock and plants. Farm children have been found to have higher levels of T helper type 1 (Th1) related cytokines compared to non-farm children, suggesting that a skewed Th1 immune response may be able to suppress the development of a Th2 response [20,21]. Immune tolerance to allergens also attributes to anti-inflammatory trained immunity, which involves epigenetic and metabolic reprogramming of innate immune cells. This is largely shaped by the microbes from the exposome, and subsequently, an immune memory is developed that can prevent allergen hypersensitivity [22]. However, the rapid increase in allergic and immunological diseases observed globally over the last few decades has led to a new theory regarding their etiology and development.

In 2017, Pothoven and Schleimer proposed a hypothesis suggesting that damage to the epithelial barrier causes abnormalities in the immune response, which in turn leads to the development of allergic diseases [23]. Climate change, Westernization of lifestyle, and urban industrialization are main sources of environmental stressors that contribute to the epithelial barrier damage and increased susceptibility to allergy development [24]. In contrast to the biodiversity hypothesis, the epithelial barrier hypothesis is based on a perpetual cycle between immunological dysfunction, chronic barrier damage, and pathogen colonization. First, external stressors such as scratching disrupt the epithelial cell barrier by inducing cellular stress that cleaves the structural elements, which leads to increased cell permeabilization [25]. Subsequently, cell damage initiates an innate inflammatory response by releasing cytokines and alarmins, and the damaged barrier allows colonization by opportunistic pathogens. Invaded pathogens reduce the microbial diversity of the local site and translocate to other subepithelial areas, whereby the overall microbial dysbiosis causes a dysfunctional immune response that is often skewed towards the Th2 response [23,26]. The environment also shapes the immune response through epigenetic regulation, which includes changes in DNA methylation and histone modification. These changes impact gene expression in epithelial or immune cells and play important roles in both the pathogenesis of and susceptibility to allergy development [27].

### 2.2. Characteristics and Functions of the Epithelial Barrier

The epithelial cell barrier lines the surface of the skin, airways, and gut and serves as the first line of defense against the external environment [28]. Prolonged exposure to environmental factors, such as chemicals, pollutants, pathogens, processed food, and synthetic materials, can pose potential risks to human health [17].

The skin, gut, and airways possess several distinguishable characteristics to prevent infection while allowing the exchange of essential elements and nutrients [29]. Epithelial cells are connected by tight junctions (TJs), adherence junctions (AJs), and structural molecules such as desmosomes. These proteins are important for maintaining barrier integrity, but TJs also limit the paracellular flux of ions and water [30]. The pulmonary airway, nasal tissue, and the gut contain a mucosal layer to trap foreign particles and pathogens, and immune cells are readily accessible to maintain homeostasis. In the pulmonary airways and sinonasal cavity, ciliated epithelial cells and goblet cells cooperate to maintain airway clearance. The cilia remove debris through mucociliary action, and goblet cells secrete mucin to trap and facilitate the clearance of toxins and pathogens [31]. The structural integrity of these barriers is primarily maintained by TJs and AJs, with allergens downregulating TJ or AJ proteins such as claudin-1, occludins, and zonula occludens, increasing barrier permeability [32,33]. Epithelial metaplasia and cilial dysfunction have also been reported.

The epithelium of the small intestine consists of five main cell types arranged in a single layer. Intestinal epithelial cells are the most abundant, with valvulae conniventes and microvilli structures to increase surface area for nutrient absorption [34]. The intestinal epithelium also contains enteroendocrine cells, goblet cells, tuft cells, and Paneth cells, all with specialized functions to maintain barrier function and homeostasis [35]. Immunological protection involves physical and biochemical barriers, where the mucus secreted from goblet cells and Paneth cells physically separates the epithelial barrier from commensal bacteria, and antimicrobial immunoglobulin A (IgA), defensins, and the continuous renewal of mucus prevent pathogen colonization [36,37]. Pathogens that override these defenses interact with pattern recognition receptors (PRRs) expressed on the surface of epithelial cells, providing additional support as part of innate immunity [38]. In addition, intestinal epithelial cells are continuously renewed through differentiation and apoptosis. Epithelial stem cells migrate from the crypts to the villi as they differentiate into mature epithelial cells. The complete duration of epithelial turnover is between three and five days, effectively minimizing potential damage from pathogens [39,40]. Commensal bacteria acquired from the environment also support the integrity of the epithelial barrier and homeostasis of the gut environment. This is achieved by competing with pathogens for nutrients and regulating metabolic activities to facilitate nutrient absorption [34]. Dysfunctional junctional proteins, epithelial cell death, metabolic stress, and the dysbiosis of the gut microbiota can increase gut epithelial permeability and lead to allergies or other inflammatory disorders [34,41].

The surface layer of the skin, the epidermis, consists of five distinct layers of keratinocytes at different stages of differentiation, with fully differentiated cells located in the stratum corneum (SC), the outermost layer. Corneocytes, dead keratinocytes in the final stage of differentiation, are organized in multiple layers in the SC. They have a flat and anucleated appearance and can be shed easily during the cell renewal process [42]. At the SC, corneocytes are bound by lipids to form the cornified envelope. These structures are linked by corneodesmosomes and are embedded in the lipid matrix. The lipid matrix is composed of ceramides, free fatty acids, and cholesterol and has natural waterproofing properties [43]. The primary function of the SC is to regulate transepithelial water loss (TEWL) and provide protection against irritants. Filaggrin proteins within the SC are essential for moisture retention, pH balance, UV protection, and immunomodulation [42]. Other homeostatic functions of the skin include prevention of mechanical injury, chemical and microbial damage, and the strict regulation of serum proteins and electrolytes [44].

**Figure 1:**
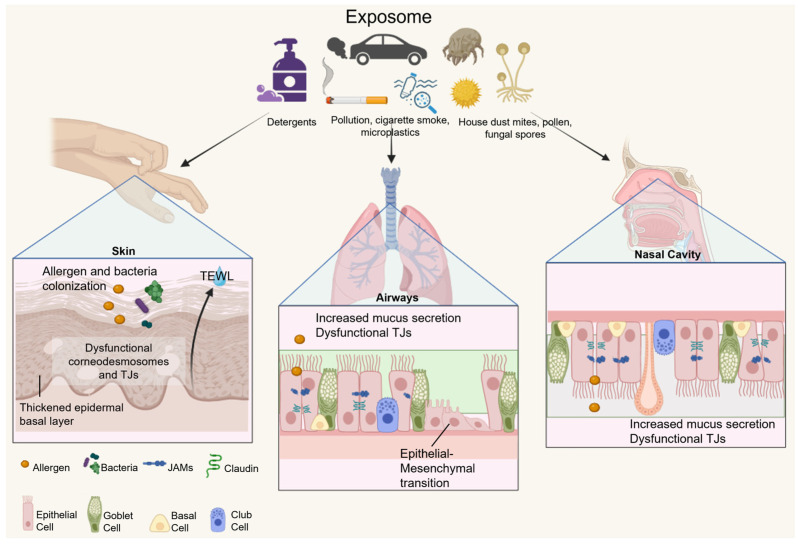
The exposome and damage to the epithelial barrier. Dysfunction of the skin (left) results in a thickened basal layer at the epidermis, increased transepithelial water loss (TEWL), and allergen and bacteria colonization. Airway dysfunction (middle) results in increased mucus production from goblet cell hyperplasia, impaired tight junctions (TJs), and epithelial–mesenchymal transition. Dysfunction of the nasal cavity leads to increased mucus secretion and impaired TJs. This figure is created by Biorender.com.

Damage caused by chronic exposure to the exposome mostly relies on tissue repair mechanisms to renew the barrier, in a process called tissue remodeling [45]. However, the continuous activation of the wound inflammatory response and regenerative inflammation can lead to metabolic changes that prevent restoration. In the skin, chronic damage causes filaggrin mutations, preventing normal keratinocyte differentiation. Desmosomes that bind keratinocytes break down, allowing the entry of allergens and bacteria and TEWL. Atopic dermatitis (AD) develops from keratinocyte dysfunction and allergen sensitization, leading to a hyperproliferation of the basal layer of the skin [44,46]. In the gut, epithelial cell renewal is based on mitochondrial metabolism, and under chronic inflammatory conditions, mitochondrial dysfunction releases reactive oxygen species (ROS) and mitochondrial DNA, contributing to epithelial damage by inducing oxidative stress [47]. Izquierdo et al. have suggested that allergy-related epithelial dysfunction results from persistent damage from both the exposome and the host-inflammatory response, leading to aberrant phenotypic and metabolic modifications. For example, airway tissue remodeling has been shown to promote goblet cell metaplasia and hyperplasia, resulting in excessive mucus secretion, reduced oxygen capacity, and increased airway obstruction [48,49]. As a result of these changes, the local environment loses its ability to restore homeostasis, and pathological mechanisms persist over time. Tissue and mucosal remodeling have been observed in the nasal polyp tissues from chronic rhinosinusitis patients and the mucosal biopsies from asthmatic patients [50]. Epithelial cells from both sites undergo epithelial–mesenchymal transition (EMT), during which cells lose epithelial characteristics and function, upregulating the expression of mesenchymal markers. This transition results in a compromised epithelial barrier function, followed by the upregulation of tumor growth factor-β expression, ultimately leading to pulmonary fibrosis [51,52]. The mechanisms of the epithelial barrier dysfunction of the skin, airways, and nasal cavity are summarized in Figure 1.

## 3. Metabolism and Cell Effector Function

### 3.1. Cellular Metabolism

Metabolism can be briefly divided into anabolic and catabolic processes. Anabolic processes use energy to build larger functional molecular units, whereas catabolism refers to the mobilization or production of energy from metabolic resources [53]. Immunometabolism can be explored from a cell-specific or crosstalk perspective, with the former focusing on the metabolic phenotypes and internal mechanisms that determine their effector function and the latter focusing on interactions between cells or organs and overall regulatory processes in the body [54]. Innate immune cells rely on sensory signaling molecules such as Toll-like receptors (TLRs) and cytokine receptors to detect external stimuli. Receptor stimulation triggers a cascade of multiple downstream signaling pathways and triggers the metabolic switch [55]. The adaptation to the overall changes in metabolic and energy demands in immune cells during cell activation is known as metabolic reprogramming [56]. This ultimately results in a shift in the cell effector functions and interactions with other cells, through changes in secretory pathways and phenotypes.

Metabolic pathways identified in immune activation include the glycolysis pathway, Krebs cycle pathway, pentose phosphate pathway, fatty acid oxidation (FAO), and amino acid metabolism [57]. Immune cells are in a quiescent state under basal conditions, and cellular metabolism is maintained at a minimal level. Their primary energy is from the tricyclic acid (TCA) cycle and oxidative phosphorylation (OXPHOS) in the mitochondria [58]. A trigger in the inflammatory response causes cell metabolism to shift from catabolism to anabolism, whereby anabolic glycolysis switches to aerobic glycolysis, or the Warburg effect. This process metabolizes glucose to lactate in the presence of oxygen, yielding products including adenosine triphosphate (ATP) and nicotinamide adenine dinucleotide hydrogen (NADH) [59]. The OXPHOS mechanism is downregulated to increase the efficiency in cell proliferation and the differentiation of immune cells [58]. In addition to the major metabolic pathways, the regulation of ROS and bioactive intermediates also serve crucial roles such as maintaining redox homeostasis and the biosynthesis of fatty acids and nucleic acids in the metabolic reprogramming of immune cells [60].

Immune cells are characterized by their specialized functions and phenotypic expression, exhibiting variations in nutritional and metabolic requirements. These requirements are influenced by changes in the extracellular and intracellular environment, such as nutrient and oxygen availability, temperature, and pH [61]. Following cell activation, the metabolic profile and outcome of immune responses are shaped by the cell-specific pathways and surrounding environmental factors, and metabolic reprogramming occurs as part of bidirectional metabolic signaling [62]. Bidirectional metabolic signaling involves the crosstalk between cellular signaling and metabolism, regulating cell effector functions, cell fate, and cell activation/quiescence. Certain key cellular mechanisms include differentiation, migration, and cytokine production [62,63]. As the metabolic demands of cells are constantly changing, immune cells acquire a high degree of plasticity for metabolic reprogramming, ensuring the efficient regulation of their respective functions in response to various stimuli [64].

### 3.2. Functions of Innate Lymphoid Cells and Eosinophils

**Figure 2:**
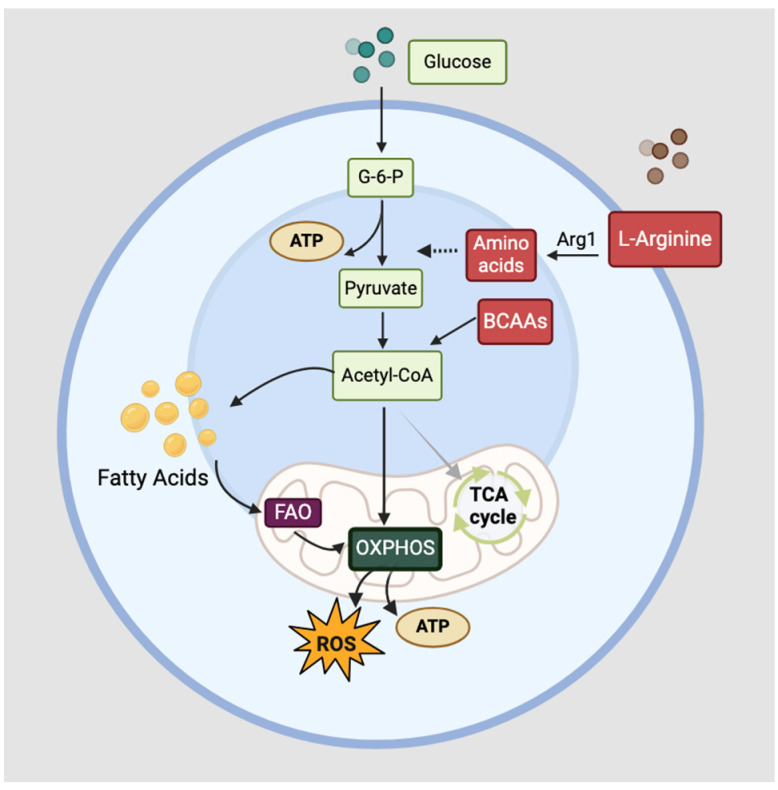
Basic metabolic regulation in ILC2s. ILC2s primarily rely on OXPHOS for energy production via FAO, both at rest or during activation. When circulating ILC2s are activated, arginine is metabolized into amino acids via the action of arginase 1 (Arg1), while BCAAs are metabolized into acetyl-CoA, contributing to the glycolytic output via OXPHOS.

Innate lymphoid cells (ILCs) and eosinophils are part of the innate immunity that participates in the late-phase inflammatory response following allergen exposure. While phenotypically similar to T helper (Th) cells, ILCs do not possess rear-ranged antigen receptors or lineage markers but can be distinguished from other cells by their surface markers IL-7 receptor alpha (IL-7Ra) and chemoattractant receptor-homologous molecule expressed on Th2 cells (CRTH2) [65]. ILCs are critical for the physical and biochemical maintenance of epithelial barriers and the regulation of inflammation. There are three subsets of ILCs: ILC1, ILC2, and ILC3, and each produces the same cytokines and functions as Th1, Th2, and Th17 cells, respectively [66]. ILC2s are a subset that is primarily involved in the type 2 immune response, which includes helminth infections and allergic inflammatory responses. ILC2s are found in the tissues, such as the mucosal layers of the gut, lungs, and mesenchymal lymph nodes, and are activated by IL-33 and IL-25 from other cells [67]. Upon activation, it produces Th2 cytokines IL-5, IL-13, and Th9 cytokine IL-9, as well as IL-6, IL-8, and CXCL10 [68]. Apart from cytokine production, it promotes eosinophil activation, mucus production, and mast cell hyperplasia during the allergic response [69]. The metabolic requirements of ILC2 vary depending on the nutrient availability at the site. Although energy can be provided through aerobic glycolysis, FAO is necessary when the local environment is deprived of oxygen or glucose, for example, at sites of parasitic infection [70]. The metabolic demands during steady state and activation strongly favor FAO to fuel OXPHOS in mice, but in both mouse and human ILC2s, arginine and branched-chain amino acid (BCAA)-mediated glycolysis also fuels OXPHOS through aerobic glycolysis in circulating ILC2s, increasing oxygen consumption and glycolytic output. Circulating ILC2s and tissue-resident ILC2s have been found to differ in their sources used in OXPHOS [71,72]. A summary of metabolic mechanisms in ILC2s is shown in Figure 2.

**Figure 3:**
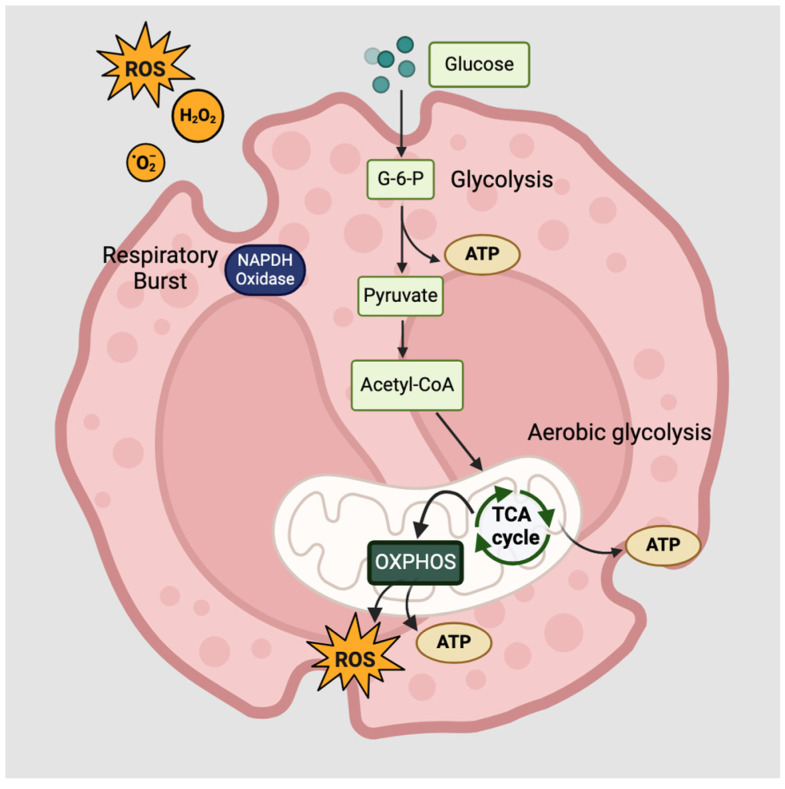
Basic metabolic regulation in eosinophils. Eosinophils have highly plastic metabolic capabilities depending on the environment and stimuli. It generates energy in the form of ATP through the process of glucose oxidation, aerobic glycolysis, or OXPHOS. OXPHOS occurs in the mitochondria, producing ROS and a high yield of ATP. Respiratory burst occurs during eosinophil activation, releasing ROS such as O_2_^−^, which is then converted into H_2_O_2_.

Eosinophils belong to the granulocyte family and are fundamental in eliminating parasites and pathogens, tissue repair, immune cell activation, and the regulation of allergic inflammation [73]. It is well established that a high eosinophil count in the blood is associated with allergic diseases. During allergic inflammation, eosinophils are activated and recruited to the local site in response to IL-5 and eotaxin, which is mainly secreted by activated ILC2s [74]. Eosinophils produce a variety of cytokines and chemokines, including IL-4, IL-5, IL-13, CCL5, and CCL11 [75]. Eosinophil degranulation occurs at the site of inflammation, releasing cytotoxic granule proteins to the surrounding environment, including major basic protein (MBP), eosinophil cationic protein (ECP), eosinophil peroxidase (EPO), and eosinophil-derived neurotoxin (EDN) [76]. This mechanism causes tissue damage, particularly in the epithelial cells, and triggers the activation of other cells to release proinflammatory products [77,78]. The metabolic activities of eosinophils are highly adaptable, with glycolysis, glucose oxidation, or OXPHOS signaling being the main energy-producing mechanisms [79]. However, this is dependent on nutrient availability and the type of stimuli, for example, IL-3-, IL-5-, or GM-CSF-stimulated eosinophils were demonstrated to upregulate in aerobic glycolysis. Mitochondrial OXPHOS not only participates in eosinophil apoptosis but also promotes the synthesis of amino acid intermediates [80]. Respiratory burst has been observed in chronic inflammatory conditions such as allergic asthma, releasing superoxide (O_2_^-^) and hydrogen peroxide (H_2_O_2_) from lysosomes to induce tissue damage [81,82]. The overall metabolic activities of eosinophils are illustrated in Figure 3.

Furthermore, eosinophils can trap allergens or pathogens by releasing a net of eosinophil DNA extracellular traps (EETs), which contain DNA and the granule protein, major basic protein [83]. The trap release process is termed eosinophil ETosis (EETosis) and is mediated by ROS production and NADPH oxidase activity. EETosis occurs during infection and inflammation to efficiently capture pathogens or allergens in these DNA traps, but it also causes and accelerates damage to the epithelial barrier [84]. In addition to the energy requirements, eosinophils are known to produce lipid mediators, which regulate lipid metabolism and are critical in the pathogenesis of allergic diseases [85].

## 4. Lipid Metabolism in Allergic Diseases

Lipid metabolism is a crucial process in metabolic reprogramming, and inflammation can lead to abnormalities in lipid production, as well as intermediate processing, within cells. Bioactive lipid mediators are directly involved in the inflammation and pathogenesis of allergies, with mast cells, ILC2s, and eosinophils being the major sources of these mediators [68,86]. Studies in allergy have demonstrated that the lipid mediators derived from these cells stimulate surrounding mucosal and epithelial tissue. Moreover, these mechanisms are responsible for the exacerbation of allergic symptoms, such as goblet cell hyperplasia, bronchoconstriction, submucosal swelling, and hyperkeratosis [87].

Arachidonic acid (AA) is an essential omega-6 fatty acid found in the phospholipid bilayer on the cell membrane [88]. AA can be processed into enzyme mediators via oxidative metabolism, including lipooxygenases (LOXs), cytochrome P450 (CYP), and cyclooxygenases (COXs) [89]. These enzyme precursors can be further metabolized into eicosanoids, such as leukotrienes (LTs) and prostaglandins (PGs). These eicosanoids are proinflammatory molecules that maintain homeostasis and modulate the adaptive immune response by mobilizing immune cells and promoting cytokine production [90,91]. LTs are classified into two groups, namely cysteinyl leukotrienes (CysLTs) and dihydroxy acids, where CysLTs include LTC_4_, LTD_4_, and LTE_4_ and dihydroxy acids include LTB_4_ [92]. Since LTE_4_ is the most abundant and stable form of all, it has a lower binding affinity for CysLT receptor 1 (CysLT_1_R) and CysLT receptor 2 (CysLT_2_R) compared to other CysLTs [93]. However, the receptors are present on most leukocytes, smooth muscle cells, and endothelial cells at varying levels of expression. CysLT_1_R is associated with bronchoconstriction in asthma, whereas CysLT_2_R is involved in pruritus in allergic skin diseases [94,95,96]. Since CysLT_1_R is more receptive to antagonists compared to CysLT_2_R, it has become a common target for allergy research in recent years. CysLT receptor 3 (CysTL_3_R), or GPR99, is expressed in the epithelial cells of airways and nasal tissue and binds specifically to LTE_4_. LTE_4_ has multiple functions in the pathogenesis of asthma, including the activation of mast cells and ILC2s, and the recruitment of eosinophils and basophils [97,98]. Although CysLTs are considered slow-produced mediators as they are only synthesized as needed, they are involved in epithelial remodeling and muscle cell hypertrophy in allergic inflammation [99].

PGs and thromboxane A2 (TXA_2_) belong to the prostanoid group and are synthesized from the further processing of PGH_2_. Formed from AAs following the enzymatic reactions of COX-1 and COX-2, the end products of PGH_2_ include TXA_2_, prostaglandin E2 (PGE_2_), prostacyclin (PGI_2_), prostaglandin D2 (PGD_2_), and prostaglandin F_2α_ (PGF_2α_) [100]. Prostanoids are produced by cells and tissues in response to stimuli that initiate acute inflammatory responses, with the goal of repair and resolution to a homeostatic state [101]. However, in allergic inflammation, PGs can exert pro-inflammatory and anti-inflammatory functions in immune cells depending on the binding receptor and the secretion duration of PGs [102]. PGD_2_ is primarily synthesized by activated mast cells and Th2 cells, which are involved in the pathogenesis of allergic inflammation. The two receptors of PGD_2_ include DP1 and CRTH2. Both receptors are expressed on eosinophils, whereas CRTH2 is expressed on Th2 cells, basophils, and ILC2s [103]. Studies have suggested that the PGD_2_ receptors serve contradictory roles in allergic inflammation. The activation of CRTH2 promotes inflammatory cytokine secretion and immune cell migration to promote allergic inflammation. In contrast, the activation of the DP1 receptor downregulates inflammation, promoting apoptosis in eosinophils and vasodilation [104,105].

Although LTs and PGs are generally proinflammatory, anti-inflammatory lipids known as specialized pro-resolving lipid mediators (SPMs) act as a counter-regulatory mechanism of acute and chronic inflammation, with innate immune cells participating in lipid mediator class switching [106]. Studies have revealed that high levels of LTs and low levels of SPMs are indicative of severe asthma or chronic obstructive pulmonary disease [107]. SPMs are synthesized by all immune cells, and they are derived from different polyunsaturated fatty acids, including lipoxins, protectins, resolvins, and maresins. Impairment in the biosynthesis of SPMs is a main factor of chronic inflammation [108].

## 5. Metabolic features of allergies

A substantial proportion of children with allergic disease are diagnosed with AD together with allergic asthma or allergic rhinitis (AR) or both. Due to its high prevalence, this phenomenon is commonly referred to as the ‘atopic triad’. One study reported that by the age of three, close to 66% of the subjects had developed one or more comorbidities, suggesting a high prevalence in children [109]. Since the pathology of these allergies is different, the metabolic regulation and cellular metabolic phenotypes will be explored, and potential patterns may be drawn.

### 5.1. Atopic Dermatitis

AD is an early-onset skin disorder characterized by intense itch, an over-proliferation of keratinocytes in the SC, and in severe cases, the formation of rough scaly lesions [110]. Common environmental factors that irritate the epidermis include direct and chronic exposure to anionic detergents, surfactants, and pollution. Chronic exposure to these chemicals alters the natural pH, promotes TEWL, and skews the skin microbiota [111]. Metabolomic studies investigating the lipidomic profile of AD patients have reported an increased ceramide shortening and a decreased synthesis of long-chain ceramides in the skin [112]. Ceramides account for around 50% of all lipids in the epidermis and can be metabolized into sphingolipids. Reduced production of ceramides or other lipids compromises barrier integrity and moisturizing properties [113]. Analysis of AD lesions in mice has shown an increase in glycolysis and the excretion of lactate. This is indicative of anaerobic glycolysis, which may contribute to short ceramide production and aberrant lipid profile [114].

For serum analysis, one study reported that AA-derived mediators LTB_4_, TXB_2_, hydroxy-octadecadienoic acids (HODEs), and hydroxy-eicosatetraenoic acids (HETEs) were significantly higher in the AD group compared to the non-AD group in children [115,116]. These mediators act as chemoattractants for leukocyte recruitment and may be involved in the itch response [117,118,119]. Sphingosine-1-phosphate (S1P) was also elevated in AD patients and was shown to correlate with disease severity [120]. However, on AD lesions in humans and canines, S1P levels were reduced [121]. S1P, produced by immune cells such as mast cells, is not only a regulator of the skin barrier, but it also regulates the mobilization of lymphocytes from the lymph nodes to the bloodstream. This is one of the key processes of inflammation which may also occur in allergic inflammation [122,123]. Furthermore, it was reported that carnitines, lactic acid, and citric acid were also elevated in the AD group. This suggests that the mitochondrial function may be impaired, and energy production may be redirected towards anaerobic glycolysis, as implicated in other studies [124]. Ma et al. found that AD adult patients presented a significant reduction in amino acid metabolites in plasma. Some examples include acetyl-L-carnitine, L-carnitine, and indole-3-acrylic acid, as well as other amino acids [125]. Since carnitines are crucial for transporting lipids to the mitochondria for energy production via beta-oxidation, these findings reflect a major shift in energy metabolism in patients with AD compared to healthy controls.

The role of eosinophils in AD has been reported mainly in CysLT production and its regulatory intracellular signaling. A study highlighted that LTC_4_ synthesized by eosinophils activates CysLT_2_R and induces fibrosis and thickening of the skin of mice with AD. It also promotes collagen production and growth factor secretion in human dermal fibroblasts [126]. Furthermore, LTC_4_ has been shown to act via the CysLT_2_R in immune cells to elicit chronic itch in the skin [96].

For ILC2s, there are limited studies on their metabolic phenotypes and pathological roles in AD. However, ILC2 levels were frequently elevated in the skin of AD patients, along with other cells such as basophils and mast cells [127]. It was found that ILC2s reside in the epidermal or upper dermal layer of the skin, termed dermal ILC2s. These cells expressed prostaglandin receptor DP2 and upregulated the molecular expression of IL-5, IL-13, and CSF2 in AD lesions but not control subjects, suggesting that ILC2s play an important role in allergic dermatitis and are likely mediated by PGs and cytokine signaling [128]. Chang et al. found that PGD_2_, released from mast cells, promoted the chemotaxis of circulating ILC2s via the PGD_2_/DP2 interaction. This finding was more significant in AD patients compared to healthy controls [129]. Although this supports the notion of lipid-mediated signaling, the mechanism of ILC2 trafficking and whether PGD_2_ acts indirectly are still unknown.

### 5.2. Allergic Asthma

Asthma is a chronic inflammatory disease that is triggered by allergic or nonallergic sources. Clinical manifestations of asthma include bronchoconstriction, dyspnea, wheezing, excessive mucus production, and cough [130]. Most asthma patients suffer from childhood-onset allergic asthma, which is linked to IgE predisposition from family history and environmental factors. Synthetic pollutants cause oxidative stress to the epithelium, where airway smooth muscle cells and resident fibroblasts undergo hyperplasia or hypertrophy [131]. This transformation upregulates TSLP production, which induces allergic inflammation by activating immune cells, from differentiating Th cells to Th2 cells, and ILC2 [132,133].

Several non-targeted metabolomic studies have shown that allergic asthma patients have presented high levels of fatty acid metabolites in their blood or sputum. A blood plasma metabolome analysis of asthmatic children has revealed a marked increase in 12(S)- and 15(S)-HETEs compared to controls [134]. Gai et al. reported that the serum of asthmatic patients with sputum containing over 3% eosinophils presented a high level of glycerophospholipid. This highlights that eosinophilic asthma, although not necessarily caused by allergy, is correlated with an increased production of lipid biomarkers [135]. Moreover, another metabolomic analysis of plasma in children with asthma showed increased markers related to the glycerophospholipid, linoleic acid, and pyrimidine pathways [136]. In sputum analysis, the metabolites of glycolysis or gluconeogenesis, glycerophospholipid, and inositolphosphate pathways were found to have the most significant changes in asthmatic patients [137]. Glycerophospholipids are part of the cell structural membrane, and there are six classes: phosphatidylcholine (PC), phosphatidylethanolamine, phosphatidylserine (PS), phosphatidylinositol, phosphatidylglycerol (PGly), and phosphatidic acid (PA) [138]. Oxidized PC is formed by oxidative stress and has been shown to trigger bronchoconstriction and increase the production of COX-2, proinflammatory cytokines, and oxylipins in allergen-challenged guinea pigs [139]. Phosphotidylcholine-specific phospholipase C (PC-PLC), an enzyme involved in the cleavage of PC, has been demonstrated to regulate eosinophil degranulation and CysLT production, indicating that it may contribute to the pathogenesis of allergy [140]. Since glycerophospholipids and their derivatives are active mediators of immune cells, these findings suggest that glycolipid metabolism plays an important role in asthma regulation.

S1P, a lipid-based derivative of sphingolipids has been detected in the bronchial alveolar lavage fluid (BALF) of asthmatic patients [141]. Eosinophil granule protein, EPO, has been found to upregulate S1P and lysophosphatidic acid (LPA), suggesting their close interaction. Functional S1P receptors, expressed by eosinophils, have been found to increase adhesion to nerve cells when bound to S1P, and this may play a role in bronchoconstriction [142]. S1P also causes airway remodeling and eosinophilic inflammation by inducing steroid resistance in airway smooth muscle cells and upregulating eosinophil chemokine eotaxin/CXCL11 [143,144].

CysLTs derived from eosinophils, mast cells, and alveolar macrophages have been directly implicated in the pathophysiology of allergic asthma. LTE_4_ and LTB_4_ are detected at higher levels in the exhaled breath condensate of asthmatic adults and children compared to controls, and both lipid mediators are inducers of bronchoconstriction [145]. LTC_4_ was found to regulate eosinophil trafficking from the intratracheal regions into paratracheal lymph nodes in mice, suggesting its role in enhancing the eosinophil chemotactic mechanisms [146]. LTE_4_ also induces bronchoconstriction to a lesser extent compared to LTC_4_ and LTD_4_ but causes aggregation of eosinophils and basophils in the bronchial submucosa of asthmatic patients [147]. Neves. et al. have shown that CysLT receptors are expressed in free eosinophil granules and that CysLTs can induce the granules to produce ECP, underscoring the potent inflammatory role of CysLTs acted in eosinophils [148].

In allergic asthma, TSLP and IL-33 from stimulated airway epithelial cells activate mast cells to secrete large quantities of PGD_2_ [149]. PGD_2_ has a short half-life, but its metabolites maintain receptor activation and mediate the effector functions of eosinophils and ILC2s. Tang et al. noted significant changes in metabolic signatures of ILC2 in mice induced with asthma, with amino acid metabolites related to sphingolipid, apoptosis, and OXPHOS signaling being the main pathways [150]. This highlights that ILC2s not only regulate cytokine signaling but also influence various metabolic pathways. Although ILC2s are a main regulator of eosinophil activity, in allergic asthma, eosinophils and their related cytokines IL-4 and IL-13 are found to regulate ILC2 reciprocally by enhancing accumulation and chemotaxis [151]. However, the metabolic crosstalk between the cells remains to be defined.

### 5.3. Allergic Rhinitis

AR is a highly prevalent allergic disorder linked to symptoms of nasal congestion, rhinorrhea, itching, and sneezing following aeroallergen exposure [152]. AR is broadly classified as intermittent or persistent rhinitis depending on the frequency of symptoms and is associated with other allergic disorders such as asthma and conjunctivitis [153]. The most common allergen source is the HDM, which can degrade claudin and TJ proteins on epithelial cells, then permeate through the weakened epithelial barrier and induce sensitization [154].

A metabolic analysis of serum samples from AR patients revealed a substantial upregulation of 15(S)-HETE, hexadecanoic acid, and a downregulation of LTD_4_. In addition to these lipid metabolites, other compounds with significant changes are part of the chemokine signaling and leukocyte transendothelial migration signaling pathways. These pathways play a role in mitochondrial dysfunction and metabolic reprogramming in the lower airways, which may increase the propensity to develop asthma [155]. Eosinophils and mast cells were found in high concentrations in the nasal turbinate tissues of AR patients. LTC_4_, LTD_4_, LTE_4_, and PGD_2_ levels were also increased in the persistent AR group compared to the intermittent group [156].

Degranulation of eosinophils in the nasal secretions was observed in individuals with AR. Both AR patients and controls expressed all S1P receptor subtypes in blood eosinophils, and one study showed that following the allergen challenge, there was a significant upregulation in most SP1 receptor subtypes in AR patients, while the control remained unchanged. The chemotaxis assay showed that S1P dose-dependently induced higher chemotactic activity in eosinophils of AR patients, illustrating the direct association between S1P and eosinophil migration [157].

ILC2s were upregulated along with eosinophils in the nasal polyps of AR patients. Cytokines from epithelial cells, IL-33 and TSLP, and CysTLs and PGD_2_ from mast cells activate ILC2s through receptor signaling [158]. This simultaneously drives eosinophil activity and contributes to allergic inflammation.

## 6. Discussion

Immunometabolism with applications in allergic diseases has been a topic of interest in recent years. In this review, we discuss the manifestations of allergy with a focus on the epithelial barrier theory, the implications of metabolic shifts in various allergic diseases, and the metabolic characteristics of eosinophils and ILC2s that are involved in the pathogenesis of allergy. Previous studies have demonstrated the importance of metabolic reprogramming in the regulation of allergic inflammation by metabolites. It has also been demonstrated that lipid mediators are fundamental to cellular function, particularly in pathways that promote allergic inflammation. Although AA-derived metabolites are not intrinsically part of the metabolome, they profoundly influence metabolic profiles in allergy. Eosinophils produce lipid bodies and chemoattractants derived from AA and other lipids, such as 5-HETE [159]. The enzyme 5-HEDH oxidizes 5-HETE to produce 5-Oxo-ETE and is found in eosinophils, neutrophils, as well as airway epithelial cells, airway smooth muscle cells, and keratinocytes [160]. Lin et al. reported that 5-Oxo-ETE, a metabolite synthesized from LTB_4_ in human neutrophils, acts as a potent chemoattractant for cells with the OXE receptor, including eosinophils and other inflammatory immune cells [161]. The presence of 5-Oxo-ETE has been documented in patients with allergic asthma and rhinitis [162].

Allergic asthma and AR patients share similarities in metabolic profiles, including metabolites likely derived from epithelial barrier dysfunction at the local allergic site, such as S1P and HETEs. Leukocytes that mediate inflammation are actively involved in the interactions with these metabolites. However, ILC2s, unlike eosinophils, may play an indirect role in mediating metabolic signals between immune cells and epithelial cells in allergic disease. Modulation of ILC2 metabolism may be able to alleviate the chronic symptoms of allergy. ILC2s mainly use lipid metabolism to fulfil the metabolic demands during allergic inflammation, but the link between small metabolite regulation and ILC2s remains an enigma.

There are many overlapping metabolites and pathways between allergic asthma, AD, and rhinitis, primarily derived from amino acids and lipids. A metabolic analysis has been conducted in 25 studies, of which 20 studies reported metabolic pathways of interest in asthma and AD, providing insights on the shared similarities in metabolomic regulation across these endotypes [163].

In the context of allergy, many metabolomic studies are at an exploratory stage of identifying potential biomarkers in clinical and preclinical samples. T cells and macrophages have been extensively investigated in allergic asthma, with some evidence into the crosstalk between immune cells and epithelial cells. For food allergy, there is currently no reliable metabolic biomarker that is specific to the gut or gut inflammation. Most food allergy metabolomic studies rely on urine, blood, saliva, or stool samples, which may not provide a comprehensive measure of the immunometabolic interplay in allergic disease [164]. Furthermore, invasive procedures such as intestinal biopsies are often not feasible in studies involving children. However, stool samples can be used to measure changes in microbial metabolites to illustrate mechanisms by which the gut microbiota shape host immunity. Microbial metabolite studies have been performed to delineate the relationship between two allergic conditions, as well as the relationship between microbial populations and allergic condition. A potential genetic link between food allergy and AD is demonstrated by filaggrin gene mutations and the increased risk of peanut allergy in a double-blind placebo-controlled food challenge [165]. The gut microbiota influence the function of Treg cells, invariant natural killer T cells, and mast cells in the regulation of food allergy and AD. Notably, short-chain fatty acids (SCFAs) derived from microbial fermentation in the gut can modulate immune hyperreactivity by inducing dermal Treg cells in mice by topical administration [166]. SCFAs also enhanced the suppressive function and upregulated the phenotypic markers of human Treg cells, which may contribute to allergic or autoimmune diseases [167]. Furthermore, poly-γ-glutamic acid (γPGA), a metabolite derived from *Bacillus subtilis*, has been shown to alleviate the allergic symptoms of asthma-challenged mice and activated Th1 cells [168]. Collectively, these findings support the notion that microbial populations at the local inflammatory site have a profound impact on the disease outcomes. The gut microbiome impacts the host immune metabolism by manipulating energy metabolism, primarily through metabolites derived from SCFA, Tryptophan, and bile acids, although their mechanisms are still under investigation [169]. Therefore, it is speculated that microbial metabolites may influence the metabolic regulation of other immune cells involved in allergic inflammation.

Current studies mainly use untargeted metabolomics analysis to identify key novel or unique biomarkers and pathways relevant to allergic disease. The main challenge of metabolomic profiling in clinical studies is the large variation in metabolic profiles and finding significant and common trends in the interactions between cells or subtypes can prove difficult, with few or no standard datasets available for reference. In addition, research on the metabolic role and regulation of ILC2s is still lacking. To better understand the metabolic role of immune cells, it is necessary to investigate their metabolic changes under oxidative stress during allergic inflammation, which requires the analysis of metabolomics at a cellular level. Future research for the metabolomics in AD at a cellular level would certainly benefit from delineating the cellular processes of all cells that partake in skin inflammation, as well as complications related to AD, such as itch and the defense against pathogens.

Biologics targeting lipid mediators, their receptors, or other metabolic markers have been tested in allergic disease. But patient responses in both clinical and preclinical studies are inconsistent. For example, some asthmatic patients treated with inhaled PGE_2_ were presented with bronchorelaxation, while some reported bronchoconstriction or coughing symptoms. But a study has shown that PGE_2_ reduced eosinophil migration in the ex vivo model, and EP2 receptor agonists used in mouse models had similar results [170]. S1P receptor agonists and antagonists are in clinical trials to treat the symptoms of inflammatory diseases. One of the drugs, Fingolimod (FTY720), has shown success in the clinical treatment of multiple sclerosis relapse and is currently being tested in preclinical studies for atopic dermatitis and allergic asthma [171,172,173]. FTY720 is a promising candidate for targeted therapy by modulating immune cells.

Recently, with the emergence of unconventional allergic mechanisms, new classifications of hypersensitivity have been defined. While type I to IV hypersensitivities are mediated by immunological components, type V and type VI hypersensitivities are mediated by tissue-derived inflammatory signals. Type V is induced by chronic tissue inflammation caused by epithelial barrier damage, leading to an aberrant immune response towards allergens. Type VI hypersensitivity is driven by the proinflammatory products released by metabolic disorders, such as obesity. Type VII hypersensitivity is directly triggered by chemicals in drugs such as non-steroidal anti-inflammatory drugs, in which CysTLs are actively involved in this mechanism. Overall, these classifications would bring new insights into allergic mechanisms and potentially drive developments toward metabolomics and inflammatory signaling [174].

In summary, it is anticipated that new treatments will take into account the complexity of mechanisms in Th2 inflammation. Overcoming the heterogeneity in allergic disease endotypes will be a challenge for targeted therapy, especially for small metabolites and receptors. The effective prediction of new target biomarkers in allergy would be essential for future therapeutic development and to provide safer and more effective alternatives to conventional drugs.

## 7. Conclusions

The process of allergic inflammation involves the manipulation of metabolites and mechanisms within cells to meet increased energy demands. In a complex interplay of inflammatory signaling, lipid metabolism is effective in mediating immune cell functions and exacerbating allergic symptoms. Immunometabolomics is now being used in both targeted and untargeted analyses of clinical specimens to identify novel biomarkers and facilitate drug discovery. Eosinophils are closely associated with lipid metabolite alterations in allergy, while ILC2s may be indirectly involved. Targeting new metabolic markers that are specific and effective against allergic inflammation will provide further understanding of disease pathogenesis and therapeutic interventions.
